# Therapeutic Effect of Bilsaan, *Sambucus nigra* Stem Exudate, on the OVA-Induced Allergic Asthma in Mice

**DOI:** 10.1155/2020/3620192

**Published:** 2020-06-14

**Authors:** Faris Alrumaihi, Ahmad Almatroudi, Khaled S. Allemailem, Arshad H. Rahmani, Arif Khan, Masood Alam Khan

**Affiliations:** ^1^Department of Medical Laboratories, College of Applied Medical Sciences, Qassim University, Buraydah51452, Saudi Arabia; ^2^Department of Basic Health Sciences, College of Applied Medical Sciences, Qassim University, Buraydah51452, Saudi Arabia

## Abstract

Asthma is characterized by the elevated level of Th2 immune responses, oxidative stress, and airway inflammation. Bilsaan, an exudate from the stem of *Sambucus nigra*, has been traditionally used in the treatment of various ailments in Saudi Arabia. Here, we investigated the therapeutic potential of Bilsaan against ovalbumin- (OVA-) induced allergic asthma in a mouse model. In order to induce allergic asthma, mice were intraperitoneally injected with alum-emulsified-OVA (20 *μ*g/mouse) on days 0, 14, and 21 that is followed by an intranasal OVA exposure from days 22 to 30. During this time, mice were orally administered with Bilsaan at the doses of 5, 10, and 25 mg/kg. The numbers of total and differential inflammatory cells and the levels of Th2 cytokines (IL-4, IL-5, and IL-13) and IgE were determined in bronchoalveolar lavage fluid (BALF). Moreover, the therapeutic effect of Bilsaan was also assessed to analyze the oxidative stress and inflammatory changes in the lung tissues. The results demonstrated that Bilsaan treatment significantly reduced the total and differential inflammatory cell count in the BALF. The BALF from the mice treated with Bilsaan showed significantly lower levels of IL-4, IL-5, IL-13, and IgE. Interestingly, a similar pattern was observed in IL-4, IL-5, and IL-13 secreted by OVA-sensitized splenocytes from the mice of various groups. Bilsaan treatment alleviated the status of oxidative stress by modulating malondialdehyde (MDA), superoxide dismutase (SOD), and catalase levels in the lung. Moreover, Bilsaan treatment reduced the infiltration of inflammatory cells, thickening of alveolar wall, and congestion in the lung tissues. The findings of the present study demonstrated an antiasthmatic effect of Bilsaan through the modulation of Th2 immune responses, inflammation, and the oxidative stress.

## 1. Introduction

Asthma influences the life of about 300 million individuals all over the world and is responsible for 250,000 deaths every year. A substantial amount of the money is spent each year in the treatment of asthmatic patients [1]. Airway inflammation is characterized by the infiltration of eosinophils, inflammatory cells, and hyperplasia of goblet cells [2]. In an allergic asthma, there has been an increased activation of predominantly Th2 cells that play a part in the progression of the disease [3]. Th2 cells secrete higher levels of IL-4, IL-5, and IL-13 cytokines that promote the inflammation and remodeling of airway cells [4]. Moreover, the presence of IL-4 or IL-13 stimulates B cells to produce IgE that binds to mast cells resulting in their degranulation [5]. The eosinophil recruitment is mediated by histamine, IL-13, and prostaglandin type 2 that are the most important players in asthma pathogenesis [6]. Besides releasing toxic proteins, eosinophils secrete many chemical mediators that promote the progression of inflammation [7]. Mast cells secrete histamine, cysteinyl leukotrienes, and prostaglandins that mediate in allergic inflammatory responses [8].

Recently, new therapeutic strategies have been suggested for asthma treatment through the modulation of the cell signaling pathways and immunologic responses. For example, antiasthmatic drug Kaempferol alleviates the airway inflammation by modulating NF-*κ*B signaling and TNF-*α*-induced lung inflammation [9, 10]. *Sambucus nigra*, commonly called Bilsaan in Saudi Arabia, and its constituents possess a wide range of therapeutic values (Table 1). Table 1 summarizes the important components of *S. nigra* and their diverse therapeutic benefits in the treatment of various ailments [11–19]. Avicenna (Ibn-e-Sina) wrote that Bilsaan dissipates the clogs and inflammation because of its hot and dry property. It cures sciatica, epilepsy, and headaches and dissolves congestion/phlegm in the chest. It improves the digestion and cures all uterine ailments [20]. Earlier reports demonstrated that *S. nigra* exhibits the antioxidant, antiviral, and antidiabetic activities [21–23]. Keeping into consideration the common use of Bilsaan to cure the respiratory diseases, we assessed the mechanism of its action in order to understand the therapeutic effects against allergic asthma.

## 2. Materials and Methods

### 2.1. Materials

Bilsaan was purchased from a registered Attar shop in Buraydah, Saudi Arabia. Ovalbumin and alum were purchased from Sigma-Aldrich (St. Louis, USA). Commercial kits to determine IL-4, IL-5, IL-13, IgE, SOD, catalase, and MDA were purchased from Abcam (Cambridge, UK).

### 2.2. Mice

Female Swiss mice (10–12 weeks of age) were obtained from the animal house facility of the College of Applied Medical Sciences, Qassim University, Buraydah, Saudi Arabia. All *in vivo* experiments were conducted by following the guidelines of the animal ethics committee of the College of Applied Medical Sciences, Qassim University.

### 2.3. Dose Standardization for Bilsaan in Mice

In order to standardize the therapeutic dose, mice were orally administered with Bilsaan at the doses of 10, 25, 50, 100, and 200 mg/kg. After seven days, the weight of mice in each group was monitored and blood was taken by retroorbital puncture to count the leukocyte numbers as described earlier [24].

### 2.4. Induction of OVA-Induced Allergic Asthma in Mice

**Figure 1:**
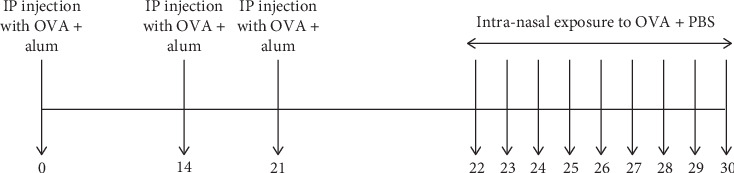
Experimental scheme for OVA-induced allergic asthma.

Allergic asthma was induced in Swiss mice by injecting each mouse with 20 *μ*g of alum-emulsified OVA on days 0, 14, and 21. Thereafter, a daily intranasal (i. n.) exposure of OVA diluted in PBS (20 *μ*l of 25 mg/ml OVA/mouse) was performed until day 30. The experimental scheme for the induction of allergic asthma is shown in Figure 1.

### 2.5. To Assess the Efficacy of Bilsaan in Treatment of Allergic Asthma

**Figure 2:**
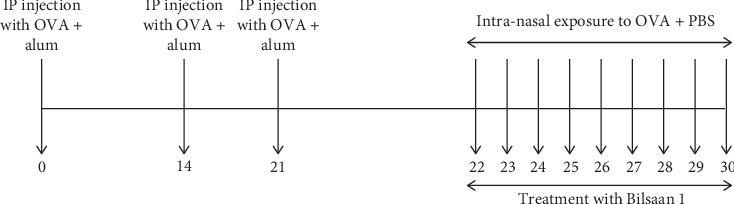
Experimental scheme for OVA-induced allergic asthma and treatment with Bilsaan.

Since the dose of Bilsaan up to 50 mg/kg was found to be safe, mice were treated with 5, 10, and 25 mg/kg doses of Bilsaan from days 21 to 30 as shown in Figure 2. Mice were divided into the following experimental groups: (1) normal mice, (2) untreated asthmatic mice, (3) Bilsaan-5 mg/kg, (4) Bilsaan-10 mg/kg, and (5) Bilsaan-25 mg/kg.

### 2.6. Determination of Cell Count and Types in BALF

Bronchoalveolar lavage fluid (BALF) was collected in 3 ml of cold PBS. The cell viability was checked by using the trypan blue. The total and differential inflammatory cell phenotypes were analyzed by an automatic cell counter. An aliquot of the BALF from the mice of each group was centrifuged at 1500 rpm for 10 minutes. A smear of BALF was made, and the slides were fixed, stained with Leishman solution for 5 minutes as described earlier [24].

### 2.7. Determination of Th2 Cytokines and IgE in BALF

The levels of IL-4, IL-5, and IL-13 were determined in the BALF by ELISA following the manufacturer's protocol [25]. The levels of total IgE and OVA-specific IgE were determined in the BALF by the IgE-specific ELISA kit (Abcam, Cambridge, UK).

### 2.8. Determination of *Ex Vivo* Cytokine Secretion by Ova-Primed Splenocytes

A single cell suspension of splenocytes was prepared as described in our earlier study [26]. The splenocytes were treated with RBC lysis buffer, and 1 × 10^6^ splenocytes/well were taken in RPMI medium supplemented with 10% FBS. The splenocytes were treated with 100 *μ*g/ml ovalbumin for 48 hours at 37°C. The supernatant was harvested in order to measure IL-4, IL-5, and IL-13 levels by ELISA as described earlier [26].

### 2.9. To Determine the Status of Oxidative Stress in the Lung Tissue

The status of the oxidative stress was determined by measuring the levels of malondialdehyde (MDA), the activities of superoxide dismutase (SOD), and catalase in the lung tissues by specific kits [27]. Briefly, a 50 mg piece of the lung tissue was homogenized in 1 ml of the lysis buffer containing 10 mM HEPES (pH 7.9), 10 mM KCl, 0.1 mM EDTA, 0.1 mM EGTA, 1 mM DTT, 0.5 mM PMSF, 2 *μ*g/ml aprotinin, and 2 *μ*g/ml leupeptin. The tissue homogenate was kept on the ice and centrifuged at 10,000 × g for 10 minutes. The supernatant was collected and stored at −80°C for further analysis.

### 2.10. To Analyze the Status of the Airway Inflammation by Histological Analysis

In order to analyze the histological changes, the lung tissues were fixed in 10% neutral-buffered formalin solution. The paraffin-embedded blocks were made, and the serial sections of 5 *μ*m thickness were slashed followed by hematoxylin and eosin (H and E) staining as described earlier [27]. The slides were examined under the light microscope (Leica, USA) at 200x magnification to observe the pathological changes.

### 2.11. Statistical Analyses

The data involving multiple groups were analyzed by one-way ANOVA following the Bonferroni post hoc test using GraphPad Prism Version 6.0 (La Jolla, CA, USA). A *P* value < 0.05 was considered significant.

## 3. Results

### 3.1. Administration of Bilsaan Did Not Induce Any Toxicity at Lower Doses

**Figure 3:**
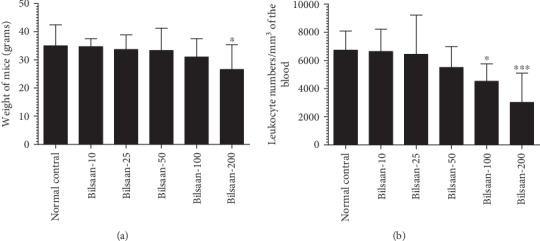
Standardization of therapeutic doses of Bilsaan in mice. Bilsaan at the doses of 10, 25, 50, 100, and 200 mg/kg were administered in mice through the oral route. Effect of Bilsaan treatment was assessed by measuring (a) weight loss and (b) leukocyte numbers. Data are expressed as mean ± SD. A *P* value < 0.05 was considered to be significant. ^∗^*P* < 0.05 and ^∗∗∗^*P* < 0.001, normal control vs. Bilsaan treatment groups.

Various doses (10, 25, 50, 100, and 200 mg/kg) of Bilsaan were orally administered in mice in order to evaluate the toxic effects in the host. Bilsaan up to a dose of 50 mg/kg was tolerated very well, whereas the treatment with higher doses of Bilsaan induced toxicity. Mice treated with Bilsaan at the highest dose of 200 mg/kg showed about 24% weight loss as compared to the mice in the normal control group (Figure 3(a)) (*P* < 0.05).

After 7 days of the treatment, the blood was taken to count the total numbers of leukocytes. Mice treated with Bilsaan at the doses of 100 and 200 mg/kg showed a significant depletion in leukocyte numbers (Figure 3(b)). The doses of Bilsaan up to 25 mg/kg were found to be quite safe, whereas a dose of 50 mg/kg caused a 19% reduction in the leukocyte number, but this reduction was insignificant as compared to leukocyte numbers in normal control mice (*P* > 0.05). Administration of Bilsaan (100 and 200 mg/kg) reduced the leukocyte numbers to 4524 ± 498 (*P* < 0.05) and 3013 ± 839 per mm^3^ (*P* < 0.001), respectively, as compared to 6729 ± 544 per mm^3^ in the blood of normal control mice (Figure 3(b)). Bilsaan caused temporarily leukopenia in mice, and once the treatment was stopped, leukocyte numbers were recovered after 12-15 days (data not shown).

### 3.2. Treatment with Bilsaan Reduced the Recruitment of Inflammatory Cells in BALF

**Figure 4:**
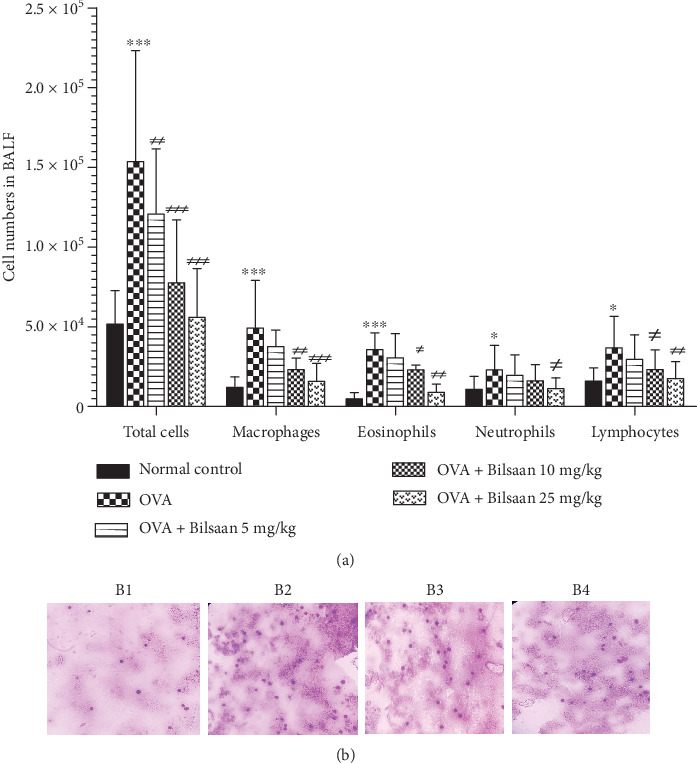
Bilsaan treatment decreases the infiltration of total and differential inflammatory cells in BALF. After 24 hours of the last dose Bilsaan treatment, BALF was collected to determine the numbers of (a) inflammatory cells. (b) BALF was spread and slides were stained with Leishman reagent. Images was taken from (B1) normal control, (B2) OVA-exposed, (B3) OVA-exposed mice treated with Bilsaan-10 mg/kg, and (B4) OVA-exposed mice treated with Bilsaan-25 mg/kg, Data are expressed as mean ± SD. A *P* value < 0.05 was considered to be significant. ∗*P* < 0.05, ^∗∗^*P* < 0.01, and ^∗∗∗^*P* < 0.001, normal control vs. OVA-exposed group. ^#^*P* < 0.05, ^##^*P* < 0.01, and ^###^*P* < 0.001, OVA-exposed vs. Bilsaan treatment groups.

To examine the effect of Bilsaan on the airway inflammation, the numbers of total and differential inflammatory cell phenotypes were counted in BALF. The total numbers of cells were found to be 153662 ± 16156 in OVA-exposed mice as compared to 51743 ± 4843 cells in the BALF of normal control mice (Figure 4(a)) (*P* < 0.001). Interestingly, the treatment with Bilsaan at the doses of 10 and 25 mg/kg reduced the total inflammatory cells to 77586 ± 9179 and 55955 ± 7105, respectively (*P* < 0.001). Similarly, the numbers of macrophages were substantially increased to 49219 ± 6952 in BALF of the OVA-exposed mice as compared to 11908 ± 1563 in normal control mice (*P* < 0.001). Bilsaan treatment at the doses of 10 and 25 mg/kg significantly reduced macrophage numbers in OVA-exposed mice (Figure 4(a)) (*P* < 0.01, *P* < 0.001). Importantly, the eosinophil count was substantially increased to 35800 ± 2430 in OVA-exposed mice as compared to 4757 ± 902 in normal control mice (*P* < 0.001), whereas treatment with Bilsaan at the doses of 10 and 25 mg/kg reduced eosinophil numbers to 22994 ± 713, 8888 ± 1199, respectively (*P* < 0.05 and *P* < 0.01, respectively). Similar patterns were noticed in the case of neutrophils and lymphocytes (Figure 4(a)).

The results of Leishman staining demonstrated the presence of high numbers of inflammatory cells in the BALF of OVA-exposed mice as compared to the cells from normal control mice (Figure 4(b), B1 and B2), whereas the numbers of inflammatory cells were found to be substantially reduced in the BALF of mice treated with Bilsaan at the doses of 10 and 25 mg/kg (Figure.  4(b), B3 and B4).

### 3.3. Bilsaan Treatment Suppressed the Production of Th2 Cytokines and IgE

**Figure 5:**
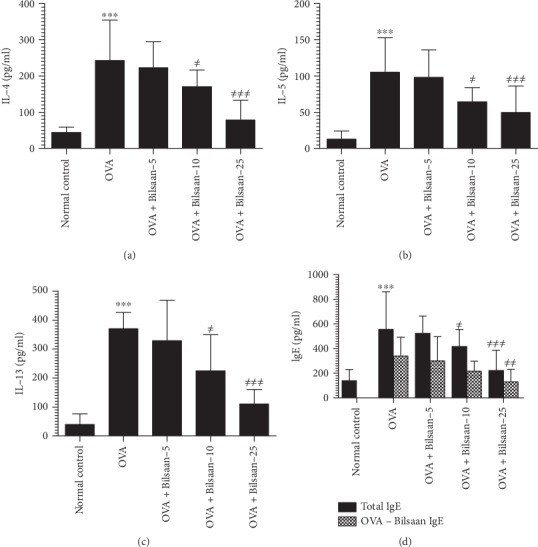
Bilsaan treatment reduced OVA-induced secretion of Th2 cytokines and IgE in BALF. BALF was collected from three mice of each group to determine (a) IL-4, (b) IL-5, (c) IL-13, and (d) IgE. Data are expressed as mean ± SE. A *P* value < 0.05 was considered to be significant. ^∗^*P* < 0.05, ^∗∗^*P* < 0.01, and ^∗∗∗^*P* < 0.001, normal control vs. OVA-exposed group. ^#^*P* < 0.05, ^##^*P* < 0.01, and ^###^*P* < 0.001, OVA-exposed vs. Bilsaan treatment groups.

Th2 cytokines play a significant role in the progression of allergic asthma. The effect of Bilsaan treatment was analyzed on the levels of IL-4, IL-5, and IL-13 in the BALF of OVA-exposed mice. There was higher level of IL-4 in BALF of OVA-exposed mice (Figure 5(a)), and it was found to be 243 ± 26 pg/ml as compared to 44 ± 3 pg/ml in normal mice (*P* < 0.001). Bilsaan treatment showed a dose-dependent effect on IL-4 production in BALF. Administration of 5, 10, and 25 mg/kg of Bilsaan reduced the IL-4 level to 223 ± 17, 171 ± 11, and 79 ± 13 pg/ml, respectively, as compared to 243 ± 26 pg/ml in OVA-exposed mice not treated with Bilsaan. Treatment with Bilsaan at the doses of 10 and 25 mg/kg, but not at a dose of 5 mg/kg, significantly reduced the IL-4 secretion (*P* < 0.05 and *P* < 0.001, respectively).

Like in IL-4, the IL-5 level was also found to be elevated in the BALF of the OVA-exposed mice (Figure 5(b)). It was found to be 105 ± 11 pg/ml compared to 13 ± 2.64 pg/ml in BALF of the normal mice (*P* < 0.001). Treatment with Bilsaan (10 and 25 mg/kg) significantly reduced the IL-5 level to 65 ± 4.4 and 50 ± 8.5 pg/ml, respectively (*P* < 0.05 and *P* < 0.01). Similarly, the level of IL-13 was also increased to 370 ± 13 pg/ml in BALF of OVA-exposed mice (Figure 5(c)) as compared to 39 ± 8.6 pg/ml in normal mice (P < 0.001). Mice in the groups treated with Bilsaan (10 and 25 mg/kg) had 225 ± 29 pg/ml and 110 ± 12 pg/ml of IL-13, which was significantly lower as compared to 370 ± 13 pg/ml in OVA-exposed untreated mice (*P* < 0.01 and *P* < 0.001, respectively).

The levels of total and OVA-specific IgE were determined in the BALF of mice untreated or treated with Bilsaan. The level of total IgE was 139 ± 21 pg/ml, whereas, no OVA-specific IgE was detected in normal mice (Figure 5(d)). The level of total-IgE and OVA-specific IgE was significantly increased to 555 ± 71 and 339 ± 36 pg/ml, respectively. They were significantly higher as compared to the OVA-specific IgE level in the mice from the normal control group (*P* < 0.001). Bilsaan at a dose of 5 mg/kg reduced the total-IgE and OVA-specific IgE to 523.66 ± 32.51 and 300 ± 45.79 pg/ml, respectively, but it was statistically insignificant (*P* > 0.05). Treatment with Bilsaan at a dose of 10 mg/kg significantly reduced the total IgE level to 416.66 ± 32.04 pg/ml (*P* < 0.05), whereas Bilsaan at a dose of 25 mg/kg caused the highest reduction in the levels of total IgE and OVA-specific IgE to 221.33 ± 38.7 and 129.33 ± 23.78 pg/ml, respectively (*P* < 0.001, *P* < 0.01).

### 3.4. Reduced Th2 Cytokine Secretion by Splenocytes from Bilsaan-Treated Mice

**Figure 6:**
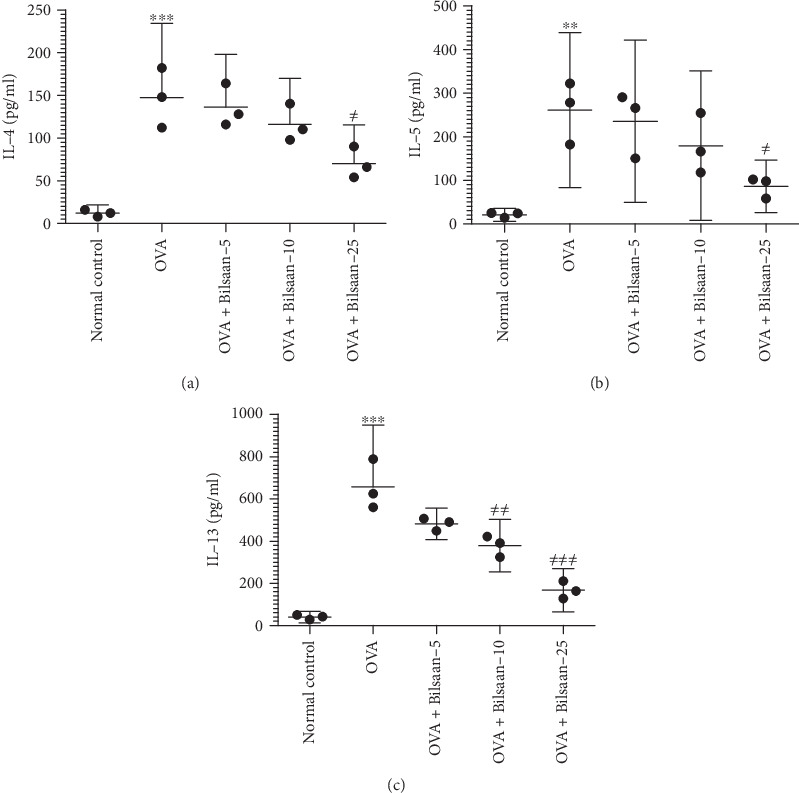
Reduced Th2 cytokine secretion by the splenocytes from Bilsaan-treated mice. The splenocytes from the mice of various experimental groups were sensitized with OVA and cultured for 48 hours. The supernatant was collected to determine (a) IL-4, (b) IL-5, and (c) IL-13. Data are expressed as mean ± SE. A *P* value < 0.05 was considered to be significant. ∗*P* < 0.05, ∗∗*P* < 0.01, and ∗∗∗*P* < 0.001, normal control vs. OVA-exposed group. ^#^*P* < 0.05, ^##^*P* < 0.01, and ^###^*P* < 0.001, OVA-exposed vs. Bilsaan treatment groups.

The Th2 cytokine pattern was analyzed in the culture supernatant of splenocytes from the Bilsaan-treated or Bilsaan-untreated mice. The splenocytes from OVA-exposed mice had substantially higher secretion of IL-4, IL-5, and IL-13 (Figures 6(a)–6(c)) as compared to those by the splenocytes from normal control mice (*P* < 0.001, *P* < 0.01, and *P* < 0.001, respectively). Bilsaan treatment at a dose of 25 mg/kg, but not at the doses of 5 and 10 mg/kg, significantly reduced IL-4 to 70 ± 18 pg/ml, as compared to 147 ± 35 pg/ml in the OVA-exposed mice (Figure 6(a)) (*P* < 0.001). The splenocytes from mice treated with Bilsaan at a dose of 25 mg/kg secreted significantly lower levels of IL-5 as compared to IL-5 secretion by the splenocytes from the OVA-exposed mice (Figure 6(b)) (*P* < 0.05). Like in IL-4 and IL-5, the secretion of IL-13 was also significantly reduced by the splenocytes from the mice treated with Bilsaan at the doses of 10 and 25 mg/kg (Figure 6(c)), as compared to IL-13 secretion by the splenocytes from OVA-exposed mice (*P* < 0.01 and *P* < 0.001, respectively).

### 3.5. Bilsaan Treatment Ameliorated the Oxidative Stress in the Lung

**Figure 7:**
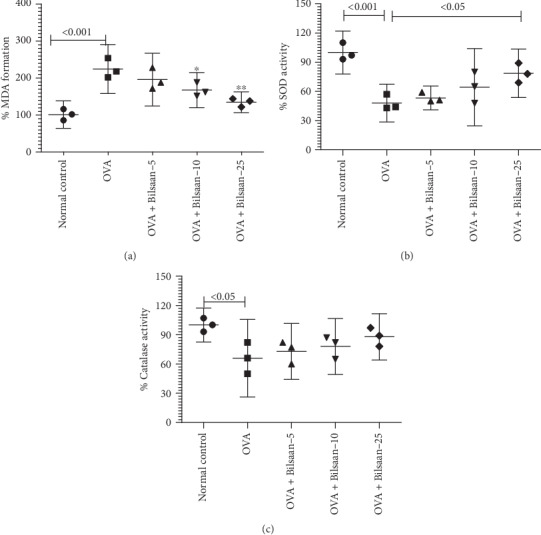
Bilsaan treatment alleviates the status of oxidative stress in BALF. BALF was collected from three mice of each group to determine (a) MDA, (b) SOD, and (c) catalase. Data are expressed as mean ± SE. A *P* value < 0.05 was considered to be significant.

The formation of MDA, a major product of the lipid peroxidation, was found to be significantly increased in the lung tissues of OVA-exposed mice as compared to that in normal mice (Figure 7(a)) (*P* < 0.001). Treatment with Bilsaan at the doses of 10 and 25 mg/kg significantly reduced the MDA level in the lung tissues of OVA-exposed mice (*P* < 0.05 and *P* < 0.01, respectively).

The activity of SOD and catalase was estimated in the lung tissue from OVA-exposed mice untreated or treated with Bilsaan (Figure 7(b)). The activity of SOD was reduced to 48% in the lung tissue of OVA-exposed mice as compared to SOD activity in the lung tissue from normal mice (*P* < 0.001). Bilsaan treatment rescued the activity of SOD in OVA-exposed mice in a dose-dependent manner (Figure 7(b)). The SOD activity was restored to 78.6% that was significantly greater as compared to 48% in OVA-exposed mice (*P* < 0.05).

Like in SOD, the activity of catalase was measured in the lung tissue of mice from various experimental groups (Figure 7(c)). The activity of catalase was reduced to 66% in the lung tissue from OVA-exposed mice which was significantly lowered as compared to normal mice (*P* < 0.05). Bilsaan treatment ameliorated the activity of catalase in OVA-exposed mice, but the recovery in catalase activity was statistically insignificant (*P* > 0.05).

### 3.6. Bilsaan Treatment Reduces the Infiltration of Inflammatory Cells in the Lung Tissues

**Figure 8:**
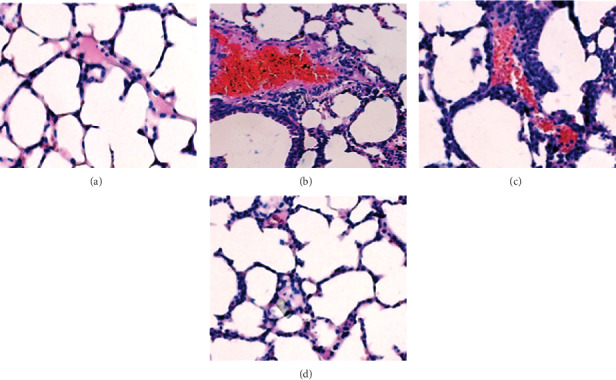
Bilsaan treatment reduces the infiltration of inflammatory cells, thickening of alveolar walls, and the congestion in the lung tissues. Histological analysis and hematoxylin and eosin (H and E) staining of the lung tissue sections from (a) normal control mice, (b) OVA-exposed mice, (c) OVA-exposed mice treated with Bilsaan-10 mg/kg, and (d) OVA-exposed mice treated with Bilsaan-25 mg/kg.

Histological analysis revealed the histological alterations in the lung tissues of OVA-exposed mice untreated or treated with Bilsaan (Figure 8). There was highly increased infiltration of inflammatory cells and the airway wall thickening in lung tissues of OVA-exposed mice as compared to that in normal control mice (Figures 8(a) and 8(b)). Moreover, the alveolar capillary was also found to be dilated and congested (Figure 8(b)). The lung tissues from the mice treated with Bilsaan at the doses of 10 and 25 mg/kg revealed the lower infiltration of inflammatory cells with reduced airway inflammation (Figures 8(c) and 8(d)).

## 4. Discussion

Allergic asthma is largely characterized by the airway inflammation, the hypersecretion of mucus, the accumulation of inflammatory cells and eosinophils, and the increased production of proinflammatory mediators and cytokines [6, 8]. *S. nigra* is one of the oldest traditional herbs that is locally being used in the treatment of dry cough, sore throat, and congestion. No studies have been performed to examine the effect of *S. nigra* against allergic asthma. The results of the present study demonstrated that Bilsaan reduced the severity of OVA-induced allergic asthma by regulating the recruitment of inflammatory cells, Th2 cytokines, and oxidative stress. Increased infiltration of eosinophils and macrophages in the BALF substantially contributes to the progression of allergic asthma [28]. Bilsaan treatment demonstrated an immunomodulatory property and significantly reduced the numbers of leukocytes in the blood and inflammatory cells in BALF.

Th2-type cytokines such as IL-4, IL-5, and IL-13 play a vital role in the progression of the allergic asthma [29]. IL-4 drives the T cells towards the Th2 type and helps B cells in antibody class switching to IgE. This is supported by the findings of the present study that showed the upsurge in OVA-specific IgE levels along with IL-4 in BALF of OVA-exposed mice. IgE activates the mast cells and eosinophil-mediated allergic asthma [29]. IL-5 is another Th2 cytokine that has a role in the progression of asthma because benralizumab, an anti-IL-5 receptor antibody, showed its efficacy in the treatment of asthma [30]. In addition to IL-4, IL-13 also contributes in the progression of allergic asthma by promoting mucus hypersecretion and eosinophils accumulation [30]. Therefore, the higher secretion of IL-4, IL-5, and IL-13 contributes substantially to inflammation in allergic asthma. Here, Bilsaan treatment effectively reduced the production of IL-4, IL-5, and IL-13 in BALF of OVA-exposed mice. Moreover, a similar pattern was found in our *ex vivo* studies, which demonstrated that the splenocytes from Bilsaan-treated mice secreted significantly lower levels of IL-4, IL-5, and IL-13 as compared to those by the splenocytes from OVA-exposed mice not treated with Bilsaan. Multiple phytochemical constituents present in *S. nigra* have their therapeutic benefits to alleviate the complications of allergic asthma. Kaempferol, one of the principal constituents in *S. nigra* (Table 1), has its therapeutic implications in the treatment of allergic asthma by inhibiting IL-4, IL-5, and IL-13 secretion [31]. Moreover, Kaempferol suppresses the goblet cell hyperplasia and mucus secretion in OVA-exposed mice [32]. Quercetin, another important component of *S. nigra*, inhibits the production of Th2 cytokines as well. Moreover, it increases the secretion of Th1 cytokines that ameliorated the pathogenesis of asthma [33]. The IgE activates the mast cells to produce inflammatory molecules that had a role in the infiltration of inflammatory cells and hypersecretion of mucus [34]. Bilsaan treatment reduced the production of OVA-specific IgE in the current model of allergic asthma.

A strong association has been suggested between the generation of oxidative stress and the progression of allergic asthma [35]. SOD, catalase, and glutathione peroxidase are important antioxidant enzymes that have potential therapeutic value in the treatment of many ailments [36]. In order to determine the status of oxidative stress, the levels of MDA formation and the activity of SOD and catalase were assessed. The increased level of MDA with reduced activity of SOD and catalase reveals the condition of oxidative stress in OVA-induced asthmatic mice, since Bilsaan contains a very high content of flavonoids and polyphenolic compounds (Table 1) that protect the SOD and catalase activity in asthmatic mice.

The infiltration of eosinophils and macrophages in BALF plays an important role in the airway hyperresponsiveness (AHR). Macrophages contribute hugely to the airway inflammatory response and lung tissue remodeling [37]. The findings of the present study demonstrated that Bilsaan treatment effectively reduced the numbers of macrophages in BALF of OVA-exposed mice. Besides, the histological analysis revealed that the lung tissues from Bilsaan-treated mice reduced the infiltration of inflammatory cells and the congestion and thickening of alveolar walls as compared to the lung tissues from the untreated OVA-exposed mice. Astragalin, one of the important constituent in *S. nigra*, has been shown to inhibit the inflammation and airway thickening [38].

In conclusion, the outcomes of the present study showed that Bilsaan treatment substantially decreased the infiltration of inflammatory cells in the BALF OVA-exposed mice. Moreover, Bilsaan treatment significantly reduced the levels of IL-4, IL-5, IL-13, and IgE. Similarly, this treatment suppressed the lung infiltration of inflammatory cells and reduced the congestion in the lung alveoli. Besides, it reduces the status of the oxidative stress by maintaining an appropriate level of antioxidant enzymes. All together, these findings substantiate that Bilsaan possesses strong anti-inflammatory, immunomodulatory, and antioxidant properties that can be very effective to treat allergic asthma.

## Figures and Tables

**Table 1:** Principal constituents of *S. nigra* and their functions.

S. N.	Constituent	Functions
1.	Kaempferol	Anti-inflammatory and antiasthmatic activity, anticancer, prevention of liver and metabolic diseases (Ren et. al, 2019).
2.	Quercetin	Anti-inflammatory and immune-stimulatory effect, anticancer, antiviral (Anand et. al, 2016)
3.	Rutin	Anti-inflammatory and antioxidant activity, nephroprotective and hepatoprotective effects (Ghorbani A, 2017).
4.	Astragalin	Anti-inflammatory and antioxidant activity, neuro- and cardioprotective, antiobesity, and antidiabetic activity (Riaz et. al, 2018).
5.	Chlorogenic acid	Anti-inflammatory and antioxidant activity, antidiabetic, anticarcinogenic, anti-inflammatory, and antiobesity (Tajik et. al, 2017).
6.	Caffeic acid	Anti-inflammatory and antioxidant, antihypertensive, antifibrosis, antiviral, and anticancer activity (Liang et. al, 2015).
7.	Protocateuchic acid	Anti-inflammatory and antioxidant activity, antimicrobial activity, anticancer activity, antiatherosclerotic activity and cardioprotective activity, hepatoprotective activity, and nephroprotective activity (Kakkar and Bais, 2014).
8.	Myricetin	Anti-inflammatory and antioxidant activity, anticancer, antiallergic, immunomodulatory, and antithrombosis (Semwal et. al, 2016).

## Data Availability

All relevant data have been provided in the manuscript.
